# Web-based information for pregnant women and new mothers with type 1 diabetes- a description of the development process

**DOI:** 10.1186/1472-6947-12-134

**Published:** 2012-11-20

**Authors:** Karolina Linden, Marie Berg, Carina Sparud-Lundin

**Affiliations:** 1 The Sahlgrenska Academy at University of Gothenburg, Institute of Health and Care Sciences, Box 457, Gothenburg, SE-405 30, Sweden; 2 University of Gothenburg Centre for Person-Centred Care (GPCC), Sahlgrenska Academy, University of Gothenburg, Box 457, Gothenburg, 405 30, Sweden

**Keywords:** Information, Pregnancy, Type 1 diabetes, Support, Website, Participatory design

## Abstract

**Background:**

This paper describes the process of developing specifically designed web-based maternity information for women with type 1 diabetes.

**Methods:**

A participatory design was used and the information was evaluated in seven stages by researchers, professional experts and users. All steps of the development process were noted in an online logbook.

**Results:**

The information developed gradually and its contents were reviewed by nurse-midwives, nurses and physicians specializing in different key areas including diabetes care, paediatrics, obstetrics and breastfeeding, a clinical dietician and mothers with type 1 diabetes. The draft was reviewed in regard to its cultural suitability and the information material was adjusted to meet quality criterions. Finally, the text was adapted for a lay audience.

**Conclusions:**

Using participatory design required time and resources, however; it proved a functional way of producing appropriate information for the target group.

## Background

Pregnant women and new mothers with type 1 diabetes need both professional and personal support [1,2]. They are at high risk for complications for both themselves and their babies, and they face a number of obstacles during the maternity period (pregnancy, labour and birth, and the first months afterwards). The increased risks include preeclampsia and complicated deliveries with foetal distress, and operational modes of delivery [3,4], foetal malformations, macrosomia, and neonatal complications [3,5-8]. Diabetes often overshadows the pregnancy [2,9] due to the struggle to maintain normal blood glucose levels 24 hours a day in order to give the child the best possible chance to be born healthy. Pregnancy for women with type 1 diabetes can be dominated by feelings of worry for the child and self-blame for not giving the child the best conditions [1,2,9]. During labour the women worry about jeopardizing their babies’ health [1]. In the early period after birth, while adapting to the maternal role and establishing breastfeeding, the women are confronted with unpredictable blood sugar levels, and many infants have hypoglycaemic episodes and other diabetes-related conditions [10].

Becoming a mother and making the transition to motherhood is typically a time of joy but also of uncertainty, trial and error [11]. Women with type 1 diabetes are exposed to risk during pregnancy, childbirth and early motherhood, and they need extra support from their partners and relatives and from health professionals during this period [1,9,10]. One essential part of giving professional support is providing readily accessible information [12]. In a Swedish study conducted in 2010, of 105 recent mothers with type 1 diabetes, 12% searched the Internet every day for information concerning pregnancy, childbirth and parenthood, 29% searched one or more times a week, and 38% searched one or more times a month [13].

Providing information through the Internet can be an effective way of supporting self-management of diabetes [14]. However, constructing patient information is not an easy task and there are a number of challenges to overcome. Traditional patient information often lacks patients’ perspectives. It outlines what the experts want the patient to know and what regime they want the patients to follow [15,16]. In contrast stands information that is designed to “empower” the reader. Even though, the idea of empowering the reader is laudable, it still reinforces the idea that the authors can control the readers’ reaction to the material. Ideally, the information material should be neutral enough for the readers to make up their own minds, this is however not easy to achieve [16]. Another great challenge is to ensure that the material is comprehendible for the readers [17].

A research program, MOtherhood and DIABetes (MODIAB), exploring and developing interventions in diabetes and childbearing [1,10], has identified needs of increased support in women with diabetes in the maternity period, i.e. pregnancy, childbirth and early motherhood. With this as a background a web-based support, MODIAB-Web, was designed, consisting of three main parts: 1) a self-care diary including a device for documenting and evaluating blood glucose levels, insulin doses, food intake, physical activities and overall well-being; 2) a forum for communication between women with type 1 diabetes in the childbearing period; and 3) specific maternity information in relation to type 1 diabetes. The objective of this paper is to describe the process of developing the specifically designed maternity information.

## Methods

### Design

A participatory design approach was used in order to develop information based on both scientific evidence and on experience-based knowledge. Participatory design can be seen as a democratic way to develop a project [18]. The discussion of values and the exploration of contradictions are important steps in the development process among different stakeholders. In regard to developing information, participatory design allows the project group to benefit from the knowledge of the participants in developing materials to empower a user. Participatory design can also help in developing a project that focuses on what the user needs rather than on what the healthcare provider thinks the user needs [19]. Different levels of participation may be used in designing a web-based health information site. These levels can range from deeply involved stakeholders who participate in every step of the design and development process to a more consultative role in which the stakeholders are asked to provide input during the design process [19]. In this project user involvement is closer to the latter description.

### Setting, participants and procedure

The project took place in two geographical areas in the western region of Sweden. Care was offered in slightly different ways in each area, which had to be taken into account when developing the information. The project was approved by the Regional Ethics Board 659–09 and is registered at ClinicalTrials.gov; id-code NCT01565824.

The project team responsible for the development of the information consisted of three persons. The first author (KL), a nurse-midwife with a background in somatic in-patient care, was responsible for developing the first drafts of the text. This author developed and wrote the text with the second author (MB), a nurse-midwife and researcher specializing in diabetes care with many years’ experience in developing care and constructing patient information. MB interacted in the text development on a detailed level. The third author (CSL), a neonatal nurse and researcher with extensive experience caring for infants born to mothers with type 1 diabetes, was in charge of the design aspects and overall appearance of the website and contributed to the text in her field of expertise. All steps of the development process were noted in an online logbook to which all members of the project team had access.

In addition several stakeholders were involved: professional experts and women representing the target group. The professional experts included clinical nurse-midwives, physicians, neonatal nurse, and a dietician. Language reviewers and editors were also included.

The information developed gradually and was thoroughly evaluated in seven stages. The development of the information itself occurred during the first five stages and the adjustment to the website in the last two stages.

### Stage 1 – Identifying the needs of information

As a first step, existing materials were identified and analyzed; this included scientific papers, the current patient information used at the hospitals, the national guidelines regarding pregnancy and diabetes care and the material concerning pregnancy and childbirth published by the Swedish Diabetes Association.

### Stage 2 – Identifying and constructing the main areas of information and it’s pats

The need of information was then determined and categorized into three main areas with separate parts. A first draft was written.

### Stage 3 – Identifying and inviting experts for revision

The project team identified the experts needed to review the information and contacted them primarily by email asking for their contribution.

### Stage 4 and 5 – Developing and reviewing the text

A final draft was written and reviewed by midwifery, medical, pediatric, breast feeding, nutrition and target group expertise. It was also reviewed in terms of its cultural appropriateness and unnecessary jargon was removed.

### Stage 6 – Design and structuring of website

The authors oversaw the design and development of the web-site. Fitting images were chosen to illustrate themes in the text and enhance lay-out and design of the web-site.

### Stage 7 - Ensuring the website met required standards

The project group ensured the website met Health on the Net Foundation’s code of conduct as well as Stvilia et. al.’s Model for Online Consumer Health Information Quality.

## Results

**Figure 1:**
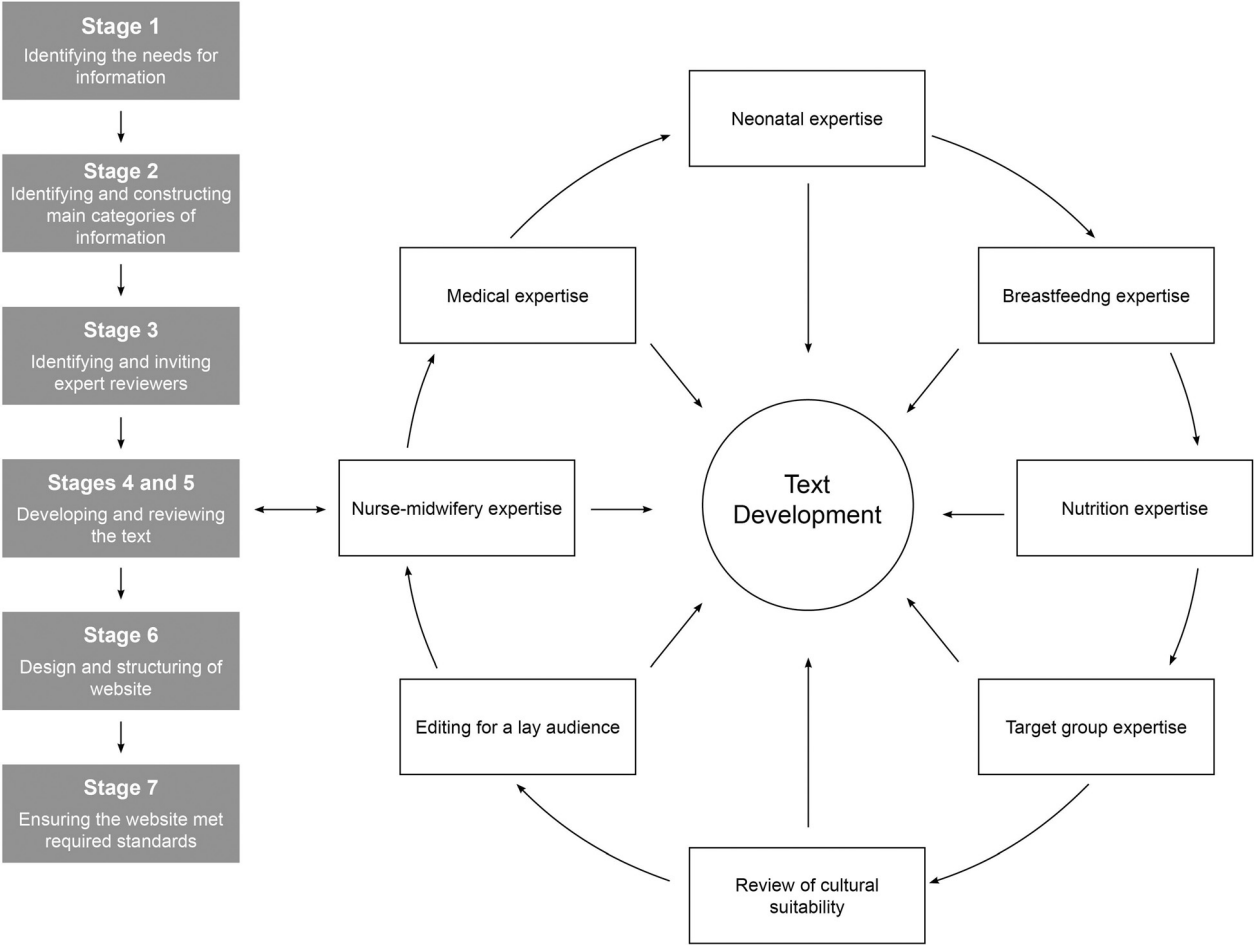
**Flowchart illustrating the development of the web**-**based information.**

An overview of the seven stages is given in Figure 1 and here follows details in each stage.

### Stage 1 - Identifying the needs for information

The first stage identified the information needs of the target group. The research team read and analysed scientific literature and patient information material on pregnancy, childbirth, parenting and type 1 diabetes. The team also examined and studied local care guidelines and national policies. The information constructed by the Swedish Diabetes Association [20] was also examined and used as a source of inspiration. From this analysis, the team identified the needs for information.

The analysis of the scientific literature revealed that women with type 1 diabetes need specific information regarding pregnancy, labour and childbirth, and what to expect of life as a new parent. General information about pregnancy, labour and breastfeeding was left out of the material as it was available on other sites. However, when the general information available was found to be lacking, in particular information regarding instrumental birth and Caesarean sections, which are more frequent among women with diabetes [3,4,21], it was decided to include sections on these topics.

### Stage 2 - Identifying and constructing the main categories of information

In stage 2, the research team used the information needs identified in stage 1 to construct the main categories of information. Three main categories were identified: being pregnant, labour and childbirth, and life as a new mother.

### Stage 3 - Identifying and inviting expert reviewers

In stage 3, which ran parallel to stage 2, the team identified experts in different fields and invited them to review the text. The experts were identified as the text was developed. In total, 18 people, both health care professionals and representatives from the target group, participated. They included five clinical nurse-midwives specializing in working with women with type 1 diabetes and five physicians specializing in medical diabetic care, obstetrics and neonatal care. There was a neonatal nurse with experience caring for babies born to mothers with type 1 diabetes, a breastfeeding specialist nurse-midwife, and a clinical dietician. There were also five mothers with type 1 diabetes, both primiparas and multiparas, who had taken part in a previous research project by the MODIAB group. One mother had a double competency, being a mother and a clinical nurse-midwife.

### Stages 4 and 5 - Developing and reviewing the text

In stages 4 and 5, the text was developed and reviewed by the project team and the experts. The development (stage 4) and review (stage 5) were part of an intertwined process and therefore both stages are described together. After each person provided input, the project team reached a consensus on what to include and to omit. The experts’ comments are described below.

### Nurse-midwifery expertise

The first draft of the text was reviewed by the clinical nurse-midwives. They reviewed the accuracy of the information as well as its appropriateness with respect to their model of care. They made suggestions, including practical advice on how to manage episodes of hypoglycaemia and on the text in the dietary information section. The text was adapted to reflect local practices and routines.

### Medical expertise

The adapted draft was reviewed by three physicians; one obstetrician and two diabetologists. Each expert reviewed the part of the text concerning his or her area of practice. All of the physicians practised in the same model of care due to a shortage of available diabetologists at one of the hospitals. They contributed recommendations of desired blood glucose levels and details on expected changes in insulin need during pregnancy and early postpartum. Consequently, the draft was revised to attain medical accurateness in both obstetric and diabetic aspects.

### Neonatal expertise

The section titled “Life as a new mother” was developed and evaluated by a neonatal nurse in collaboration with a neonatologist. The text was further adapted to local guidelines, and changes were made until a consensus was reached between the neonatal nurse and neonatologist.

### Breastfeeding expertise

The section titled “Breastfeeding” was partly written by a midwife specializing in human lactation. It was reviewed by the members of the project group as well as the neonatologist and the obstetrician. Changes were made to the text in regard to breastfeeding advice and how the infant’s health affects early breastfeeding.

### Nutrition expertise

The accuracy of the dietary information was assessed by a clinical dietician. Following her review, changes were made to the text about the use of artificial sweeteners during pregnancy and when breastfeeding.

### Target group expertise

After ensuring that the written information was correct, the latest draft of the information was sent to the five mothers. Their review of the text and comments on its contents, tone and usefulness to them provided invaluable input. Changes were made based on their suggestions. Almost all of the headings were changed, as one mother pointed out that the overall tone of the text was too focused on complications and possible hardships. Consequently, parts of the text were rephrased and the order of the sections changed to achieve a more positive tone that focused primarily on the pregnancy and secondarily on diabetes.

The mothers validated the information by acknowledging its relevance. One commented: *“Yes, it was exactly like this …”* and stated that *“I would have loved to have read the texts if they were available (when I was pregnant) …” “…They are especially for us”.* The women had different opinions on the section regarding healthy eating. One mother thought it was *“bang on”* whilst another stated that “*a mother-to-be with type 1 diabetes already knows this”.* This is in line with the existing research [22-24]; pregnant women with type 1 diabetes have different information needs, some being quite knowledgeable about their diabetes and others needing basic information. After discussing the information on healthy eating, the project group decided to keep it as some women had found it valuable, and those who had found it excessive could skip this section.

### Review of cultural suitability

After the reviews by the experts and the target group, all of the written information was assessed in regard to its cultural suitability and readability. This assessment was conducted by three skilled professionals: a journalist and director of a patient information website, a medical secretary, and a nurse-midwife with a master’s degree. To evaluate the text they were provided with the criteria of the Suitability Assessment of Materials [25]. The instrument was partly used and the text was reviewed in regard to its content, literacy demand, learning stimulation and motivation as well as its cultural appropriateness. Some changes to language and context were made and some parts were removed as they were found to be unnecessarily detailed. For example, the section on instrumental birth was reworked as it was too specific.

### Editing for a lay audience

The text was professionally edited to remove unnecessary jargon and to ensure that it could be understood by its intended readers. The research team hired a commercial company specializing in popular science editing for this purpose. They changed the order of the paragraphs and reorganized the parts in a way that further enhanced the text, making it more comprehensible. Making these changes improved the flow and readability of the text. As a last step, the first and second authors reviewed the text to make sure that no meanings had been changed unintentionally and that no significant information had been removed.

### Stage 6 - Design and structuring of website

In stage 6, the website was designed and laid out by web developers. The researchers had input on the design. An overview of the final information with categories and content is presented in Table 1.

**Table 1:** An overview of the information provided on the website

**Categories**	**Content**	**To cover**
Being Pregnant	General introduction to the theme	Most aspects in regards to the changes in one’s body when pregnant and living with type 1 diabetes.
	Glycaemic control	
	Nausea and type 1 diabetes	Aspects of care and monitoring during the pregnancy.
	Blood sugar and its effect of the foetus	
		Aspects of the pregnancy’s effect on one’s diabetes.
	Pregnancy monitoring for women with type 1 diabetes	
		Practical suggestions on how to adapt to being pregnant and living with type 1 diabetes.
	The care team	
	Hereditary factors	
	The increased need for insulin	
	Types of insulin and other medications during pregnancy	
	Hypoglycaemia and insulin coma	
	Physical activity for pregnant women with type 1 diabetes	
	Good habits for achieving normoglycaemia	
	Dietary information for pregnant women with type 1 diabetes	
Labor and Childbirth	General introduction of the theme	Specific information in regards to living with type 1 diabetes and giving birth.
	Vaginal childbirth	
	Vacuum-assisted vaginal delivery	General information about instrumented birth and Caesarean- section as this information was found to be lacking in the general web-pages.
	Emergency C-section	
	Scheduled C-section	
Life as a New Mother	General introduction of the theme	Specific situation that can occur in regards to the baby’s and mother’s health after delivery.
	The baby’s wellbeing after birth	
	Early breast feeding	The needs of women with type 1 diabetes after becoming mothers.
	Blood sugar levels after giving birth	
	Life as a new mother	To strengthen women with type 1 diabetes in breast feeding.

In order to avoid overwhelming the user with information, the parts of the categories have been kept independent of each other, and they can be read sequentially or separately as the user wishes. This allows the user to select only the sections of desired information.

**Figure 2:**
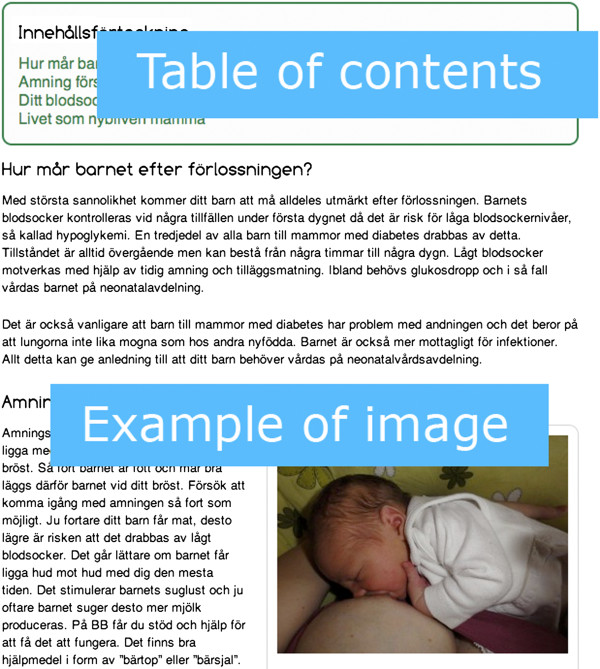
Screenshot of an example of developed information.

In this stage, images and links were added to enhance and complement the understanding of the text. Fitting images were chosen by the first and the third authors, who checked copyrights and acquired permissions needed. Links were also provided to evidence-based sites with general information about pregnancy, labour, breastfeeding and diabetes. The structure of the information on the website was assessed by three pregnant women having diabetes type 1. An example of the design of the information part of the website is given in Figure 2.

### Stage 7 - Ensuring the website met required standards

In stage 7, the first and third authors ensured that the website met the Health on the Net (HON) code of conduct [26]. The code was developed by the HON Foundation, a non-governmental organization, and is an ethical code for presenting health information. It consist of eight principles: authoritative (give qualifications of authors), complementarity (information to support, not replace), privacy (respect the privacy of site users), attribution (cite the sources and dates of medical information), justifiability (justification of claims / balanced and objective claims), transparency (accessibility, provide valid contact details), financial disclosure (provide details of funding) and advertising policy (clearly distinguish advertising from editorial content). If the website honours these principles, the site owner can apply for HON code certification to put on the website. This service is free of charge and helps readers recognize the health information given as accurate and trustworthy.

Stvilia et al. have developed a Model for Online Consumer Health Information Quality [27]. The model consists of five criteria: accuracy (ensuring credibility and reliability), completeness (ensuring clarity), authority (ensuring verifiability), usefulness (ensuring ease of use, objectivity and utility) and accessibility (ensuring consistency, cohesiveness and volatility). All five criteria need to be met for a website to be recognized as of high quality.

The research group chose to apply both criteria after the information was developed because it found it important to allow the design process to be as free as possible. Being too focused on fulfilling these criteria in the development of the site might have hampered creativity during construction of the website. Only minor adjustments had to be made in order to fulfil the criteria, such as providing full qualifications of all of the expert reviewers.

## Discussion and conclusion

### Discussion

Developing information based on scientific evidence and on experience-based professional knowledge through a participatory design was functional, although time-consuming. The process benefited greatly from having a heterogeneous group of experts assessing the materials independently. Having access to a group consisting of experts in medical care, popular science editing, and mothers with type 1 diabetes who had lived experience of pregnancy, childbirth and early motherhood proved indispensable because the final text would not have been as thorough and well thought out without their input.

Reaching a consensus on what to include or omit after each expert’s review and revision required time for reflection on the part of the research group. Using participatory design proved an effective way of ensuring that the information provided reflected what the women wanted to read about [19]. Having input from both professionals and women/ mothers who had experience with diabetes and childbearing provided insights that were invaluable to the project team. Getting the mothers’ opinions was undoubtedly the most important part of the review process, a step that could not have been left out. Their comments allowed the research team to change the content, structure and tone of the text to better suit their needs, as is vital in evidence-based information [28,29]. Since pregnant women and women who have recently given birth are in transition to their new role as mothers [11], it was important that the information reflect the various stages of their journey.

Developing specialized information is a challenge. There is often a gap between what the health professionals want the reader to know and what the reader is expecting to read. In traditional patient information for patients with diabetes the focus has often been on getting the patient to comply with the physician’s prescribed treatment [15]. In moving towards a model of shared decision making, evidence-based information is necessary for making informed choices [28,30]. There appears to be a lack of theory on how to measure whether patient information is comprehensible or not [17]. In this project the research team consulted a commercial company specialized in popular science editing to gain insight from professionals not biased by pre-understandings. This was helpful, but not essential. If the team had not had the insights from the target group, it probably would have missed the mark in tone and meaning. The same content may differ in perceived meaning depending on the tone of the paragraphs and headings, a valuable insight. This highlights the importance of involving members of the target group and not only skilled professionals in text development [29].

When designing the information to suit a web setting a few considerations had to be taken into account. One is that the content must meet appropriate standards. As novices in web-based patient information development, the research team found that the Model for Online Consumer Health Information Quality [27] and Health on the Net Foundation’s code of conduct [26] helped ensure that the website met an acceptable standard. The design and layout of a website also affects users’ perceptions of credibility [28,31,32]. The recognition of well-known brands or symbols on a website help users of the site determine whether the information given is credible [32].

### Limitations

This research project of developing information specifically designed for pregnant women and new mothers with type 1 diabetes had some limitations. First, it is only available in Swedish which excludes all women who do not comprehend written Swedish. When implemented in usual care the web support needs to be translated to common foreign languages. In this process, women with diabetes using the specific language, needs to be involved. Second, it is limited to the geographical areas in which it was developed. It could be adapted to suit a national target group and needs to be reassessed, a process that the research team has begun by including experts from other parts of Sweden to participate. Third, the physicians who reviewed the information all worked at the same hospital, so local bias may affect the review. This is also being corrected as physicians from other hospitals are taking part in the reassessment. Fourth, the information lacks a section written especially for the women’s partners, relatives and friends who are likely to also access the website.

It was important to the project group that the information reflect what the women needed. One way to ensure this would have been to invite women from the target group to participate in the initial planning of the project and in reviewing previous research. While this can be done successfully, it would require a great deal of effort on the part of participants [33]. In this project, the insights provided from previous research of the target group [1,2,10] were used as a foundation.

It is difficult to measure whether the text developed is culturally appropriate or not. The Suitability Assessment of Materials scale provides some guidelines on how this can be done [25] but the instrument was not developed solely for this purpose. A developed version of the Suitability Assessment of Materials scale where comprehensibility was added is available [34]. The authors were not aware of this version when the project took place. However, there appears to be a lack of validated instruments intended to determine cultural appropriateness [28]. In this project, the research team benefited from having input from several and complementary professionals in developing the information text. Even though only three professionals were specifically asked to review cultural appropriateness, all participating reviewers, including members of the target group, participated in this assessment in some way.

### Conclusions

Development of specialized information is one aspect of providing professional support. Using participatory design proved to be a functional way of developing evidence-based information. Based on the experience gained from this project, the research team found it important to allow enough time and provide enough resources to permit the project to evolve at an unhurried pace. Getting input from different and relevant professionals not only allowed the text to evolve but also required the researchers to take time for reflection. Asking for the patients’ views of accuracy and relevance was the most important step. Using participatory design helped ensure that the information on the website reflected what the women wanted to read, not just what the health professionals wanted the women to know. The comments from the women in the target group also ensured that the text appropriately reflected their transition to motherhood. More research that explores the use of participatory design in developing information is needed. There appears to be a need for validated instruments that measure cultural suitability.

## Competing interests

The authors declare that they have no competing interests.

## Author’s contributions

The study was designed by MB and CSL. KL collected data and wrote the initial draft of manuscript. MB and CSL carefully and repeatedly reviewed and edited the manuscript. All authors approved the final manuscript.

## Pre-publication history

The pre-publication history for this paper can be accessed here:

http://www.biomedcentral.com/1472-6947/12/134/prepub
